# A hybrid Bi-LSTM and RBM approach for advanced underwater object detection

**DOI:** 10.1371/journal.pone.0313708

**Published:** 2024-11-22

**Authors:** Manimurugan S., Karthikeyan P., Narmatha C., Majed M. Aborokbah, Anand Paul, Subramaniam Ganesan, Rajendran T., Mohammad Ammad-Uddin

**Affiliations:** 1 Faculty of Computers and Information Technology, University of Tabuk, Tabuk City, Kingdom of Saudi Arabia; 2 Saveetha School of Engineering, Saveetha Institute of Medical and Technical Sciences, Chennai, India; 3 School of Computer Science & Engineering, RV University, Bengaluru, India; 4 Department of Biostatistics and Data Science, The School of Public Health, Louisiana State University Health Sciences Center, New Orleans, Louisiana, United States of America; 5 Department of Electrical and Computer Engineering, Oakland University, Rochester, Rochester, Michigan, United States of America; 6 Department of Sustainable Engineering, Saveetha School of Engineering, Saveetha Institute of Medical and Technical Sciences, Chennai, India; Chunghwa Telecom Co. Ltd., TAIWAN

## Abstract

This research addresses the imperative need for efficient underwater exploration in the domain of deep-sea resource development, highlighting the importance of autonomous operations to mitigate the challenges posed by high-stress underwater environments. The proposed approach introduces a hybrid model for Underwater Object Detection (UOD), combining Bi-directional Long Short-Term Memory (Bi-LSTM) with a Restricted Boltzmann Machine (RBM). Bi-LSTM excels at capturing long-term dependencies and processing sequences bidirectionally to enhance comprehension of both past and future contexts. The model benefits from effective feature learning, aided by RBMs that enable the extraction of hierarchical and abstract representations. Additionally, this architecture handles variable-length sequences, mitigates the vanishing gradient problem, and achieves enhanced significance by capturing complex patterns in the data. Comprehensive evaluations on brackish, and URPC 2020 datasets demonstrate superior performance, with the BiLSTM-RBM model showcasing notable accuracies, such as big fish 98.5 for the big fish object in the brackish dataset and 98 for the star fish object in the URPC dataset. Overall, these findings underscore the BiLSTM-RBM model’s suitability for UOD, positioning it as a robust solution for effective underwater object detection in challenging underwater environments.

## 1. Introduction

Detecting and classifying submerged objects represent a fundamental component within underwater applications across various domains, including industrial security and scientific investigations within marine biology and archaeology. In contrast to terrestrial tasks, the process of detecting and classifying objects underwater differs in consideration of the influence of water density on the limitation of light penetration. Moreover, the deficiency in transparency of the aquatic environment, the level of water turbidity, the depth of the surface, and the prevailing surface circumstances are influential factors that contribute to the quality of imagery obtained. Simultaneously, dim lighting and excessive noise present significant challenges in comprehending marine video or image analytics [1]. Moreover, an aggravating factor contributing to this issue is the intrinsic under-water distortion, which involves light scattering and absorption. This phenomenon reduces contrast, diminished colour rendition, uneven illumination, and blurred imagery [2]. The intricacy of computer vision activities, such as detection and recognition in an underwater environment, surpasses that of open-air surroundings. Identifying and localizing subaquatic diminutive entities poses a formidable task [3]. Improving the identification efficacy of small targets would critically contribute to achieving optimal recognition precision. In the context of down-sampling procedures that involve deep neural networks, a noteworthy finding pertains to the gradual vanishing or reduction of feature data about smaller targets. This phenomenon occurs concurrently with an expansion in the count of network layers. Hence, UOD and classification require specialized technical measures and image-processing algorithms.

Numerous scholars have suggested effective techniques for merging features or extracting superficial characteristics to identify targets of different scales [4]. Numerous scholars have highlighted the importance of constructing intricate routes to integrate functionalities originating from discrete convolutional layers, kernels, and cohorts. The notable expansion of Deep Convolutional Neural Networks (DCNN) and the considerable enhancement in General Processing Unit (GPU) computing power constitute the primary drivers of the expedited advancement in Computer Vision (CV)-based object detection. Concerning this matter, it is notable for underscoring the advancement of DL (Deep Learning) from Machine Learning (ML) and the distinctive dissimilarity thereof [5]. ML is an artificial intelligence (AI) subfield focused primarily on acquiring knowledge from trial data or examples by identifying patterns. Recognizing objects within the context of DL architecture is commonly known as a process that involves accurately labelling various discrete objects within an image frame. This task also involves forecasting bounding boxes concerning these particular objects with an enhanced probability [6–8]. The limitation of underwater vision systems is their instability when detecting objects beneath the water’s surface. The images captured by cameras in underwater environments are prone to various forms of degradation, including intensity degeneration, colour distortion, and haze effects. Numerous techniques for enhancing or restoring underwater images have been incorporated as a pre-processing step before feature extraction in object detection models to facilitate clear and distinguishable underwater image analysis. If image pre-processing techniques fail to account for the optical properties of the underwater environment. In that case, the incorrect labelling of emerging noise sources and erroneous colouration may lead to misclassifications of the targeted objects. The rising need for vision-based applications has amplified the significance of camera-based object detection techniques in underwater environments [9]. The present investigation extracted empirical data from diverse types of water sources like bubble-containing water, turbid water, and effluent-free water.

### 1.1. Scope of the research

The incorporation of convolutional neural networks (CNNs) has resulted in improved recognition accuracy. However, the employment of extensive network weights has also led to an increase in computational costs. The current conventional CNN and Bi-LSTM-RBMs models have yet to demonstrate significant improvements in achieving higher accuracy and dropping the complexity and cost of the existing UOD systems. The present study advances an innovative DL-based technique for UOD. This approach utilizes RBF-based K-means clustering in conjunction with Bi-LSTM networks. The hybrid model exhibits a superior level of accuracy in comparison to its single counterpart. To enhance the accuracy and precision of this research, a hybrid technique is proposed to detect and track objects.

### 1.2. Research motivation

The critical need to advance autonomous underwater exploration and object detection in challenging deep-sea environments motivates this research. Existing literature has demonstrated significant efforts in utilizing various deep-learning techniques to enhance accuracy in underwater object detection. However, a substantial research gap persists in addressing the dynamic and variable nature of underwater scenarios, where temporal dependencies and complex patterns play a crucial role. The proposed hybrid Bi-LSTM and RBM approach is motivated by its potential to provide a holistic solution by combining the strengths of sequence processing and hierarchical feature learning. Through this approach, the research aims to significantly enhance the precision of detecting objects in underwater environments, contributing to the development of more robust and efficient autonomous systems for deep-sea resource exploration and underwater tasks.

This study introduces a novel framework for UOD, which involves implementing a selection approach for essential sequence segments. This method utilizes the Radial Based Function Network (RBFN) to measure segment similarities within clustered groups. The present study employs the Short-Time Fourier Transform (STFT) algorithm to convert the selected sequence into a frame. The resulting frame is subsequently subjected to processing by the CNN model, which facilitates the extraction of crucial discriminative features that effectively highlight the salience of the underwater entity. Additionally, discerning characteristics are extracted at a high level from chosen segments by employing the "ResNet-101’s FC-1000" layers [10] framework.

The main contributions of the research paper are summarized as follows.

- The paper proposes a hybrid UOD model that combines deep Bi-LSTM with a Restricted Boltzmann Machine (RBM). This hybrid approach aims to improve the accuracy of object detection in underwater scenarios.
- The fusion approach proposed herein presents a marked enhancement to the precision of the model in detecting imprecise and diminutive targets beneath the surface of the water. This strategic combination holds substantial promise as an efficacious solution for identifying targets in submerged environments.
- The proposed system is evaluated on brackish, and URPC 2020. The evaluation aims to advance recognition accuracy and reduce processing time.

The rest of the paper is organized as: Section 2 analyzes the related works. Section 3 elucidates the research methodology. Section 4 presents the evaluation, result analysis and discussion, while Section 5 details the concluding remarks.

## 2. Related works

The investigation of object detection within underwater environments is being followed to establish a connection with the core principles of HSV segmentation, which rely on colour categorization. Ji et al. [9] performed a work on the effects of social media on adolescent behaviour. The development of a self-sufficient aquatic robot capable of undertaking underwater tasks such as object, collision avoidance, and identification has been achieved. The study aimed to design a computerized model for robotic fish utilizing state-of-the-art software called Solid Works. The system facilitated the direct export of stereolithography (STL) files to MakerBot, a specialized 3D printing machine, to build the robotic fish parts utilizing thermoplastic polylactic acid polymer. Forward-looking sonar needs more automated object classification, primarily due to the scarcity of effective samples and inadequate signal-to-noise ratios (SNR). In their work, Cai et al. [11] introduced a method for detecting underwater objects within the context of weakly supervised learning. The approach involved the simultaneous training of two DL detectors, where selecting optimal samples with minimal noise informed a reciprocal teaching process between the two detectors. The base of every identifier was You Only Look Once (YOLO) v5, which attained a balance between precision and efficiency. The model was evaluated on the URPC2021 data set and has demonstrated exceptional performance, thereby attaining better results. When correlated to the traditional Yolov5 algorithm, including a dual training strategy results in a noteworthy enhancement of recognition accuracy, with an observed improvement of 10%. The identification process of UOD necessitated manual intervention or post-processing onshore, leading to a notable impact on the immediacy and real-time execution of underwater tasks. The recognition and classification accuracy are susceptible to impairment due to indistinct image boundaries and multiple instances of noise in the image. These limitations arise from the intricacies of sound propagation within aqueous environments and the associated properties of sound waves.

Fenglei et al. [12] developed a method utilizing a CNN model to address the issue of inadequately illuminated underwater images. In conjunction with image processing techniques, deep CNN was applied to classify and detect underwater objects. The enhancement of the deep CNN architecture was achieved through dual modes of refinement. The initial technique involved down-sampling a 26 × 26 feature map by implementing a 1 × 1 convolutional kernel to achieve a 13×13 output. In the following process, a layer of down sampling was incorporated before the convolutional layers. The following scheme exhibited superiority over YOLO V3, Faster RCNN, and Fast RCNN. The methodologies mentioned above were predicated upon a solitary model. Nonetheless, conventional techniques for detecting objects in underwater imagery failed to effectively utilize the profound attributes of sonar imagery to make informed decisions. Simultaneously, it was common to possess a deficiency in strength and capacity for generalization.

Krishnan et al. [13] studied the tracking and detection of underwater objects by utilizing the Hybridization of Deep CNN for UOD (HDCNN-UOD) model. The research employed brackish, UOT32, and URPC 2020 data sets as benchmark datasets. In addition, an integration of dual DL methods, specifically the EfficientNet and RetinaNet models, was employed as feature extraction tools. Additionally, predicting the bounding box was executed using the SVR (Support Vector Regression) approach, which was subsequently complemented by the KELM (Kernel Extreme Learning Machine) approach. The concept of the study was represented through the implementation of an SVR approach for bounding box regression and the utilization of a fusion-based feature extraction method. The investigation found that the model attained the most elevated degree of precision of 94.85% for the ’Crab’ object when utilizing the brackish dataset. Additionally, when using the URPC dataset, the HDCNN-UOD model achieved a peak precision of 88.34% for the ’Scallop’ object, surpassing the results obtained by the T-YOLO v4 model. Therefore, according to the findings, the HDCNN-UOD method demonstrated more suitability in object tracking and detection applications. The utilization of this particular tool has been limited to minor applications, with suboptimal performance in monitoring and tracking capabilities.

Ning Jiang et al. [14] employed DL to optimize the detection of underwater images and developed a markers dataset. The authors have posited that Image Processing Algorithms exhibited a high degree of efficacy in detecting and recognizing airborne markers. The complex underwater imaging environment posed a significant challenge to optical vision systems, resulting in image degradation. The need for object recognition information in degraded underwater imagery poses significant challenges to the detection and identification. The advancements in high-tech underwater imaging have resulted in improved imagery quality. However, challenges such as declining, slight contrast, and blurred features persist. The domain of ocean exploration was confronted with a multitude of formidable challenges. The YOLO v3-based object identification model was trained on aerial marker images, utilizing a DL network, and subsequently fine-tuned for underwater markers. The target marker was identified through the implementation of image processing techniques. The accuracy achieved by YOLOv3 in the context of dark underwater imagery was considerably higher at 92.9%, in contrast to the comparatively lower accuracy of 75.4%.

Han et al. [15] employed a fusion approach utilizing the max-RGB and shades of grey methods to augment underwater vision. Additionally, a CNN method was proposed to address the issue of diminished illumination in underwater images, wherein the CNN was trained to establish a mapping relationship capable of computing the corresponding illumination map. Following image processing techniques, a deep CNN model has been implemented for underwater object classification and detection. Because of the distinct attributes of underwater vision, two enhanced modifications have been incorporated into the deep CNN structure to improve its efficacy. This investigation conducted a comparative analysis between the Fast RCNN, Faster RCNN, and the original YOLO V3 models in conjunction with Scheme 2. The results of this analysis unequivocally demonstrated that Scheme 2 exhibited superior performance in detecting submerged objects. The detection rate registered at approximately 50 frames/second, while the mean average precision (mAP) measured approximately 90%. The application of the program in an underwater robot has yielded noteworthy outcomes. Specifically, real-time detection results have demonstrated that the program facilitates precise and expeditious detection and classification, enabling the robot to conduct underwater operations efficiently. Chen et al. [16] introduced SWIPENet (Sample-Weighed Hypernetwork) as a potential solution for small item identification in underwater environments. In addition, a re-weighting method known as IMA (Inverted Multi-Class Adaboost) was presented for mitigating any undesirable noise in the system. This approach represented a valuable contribution to the field of subaquatic object recognition. The findings derived from experiments on the URPC2018 and URPC2017 datasets indicated that the SWIPENet+IMA model outperformed many currently available object detection techniques. The authors have asserted that utilizing an ensemble of M DNNs results in a time density for the approach that was M-fold greater than that of a solitary model.

Wang et al. [17] developed a YOLO-based technique for detecting underwater objects in underwater images. This article presented an enhanced detection technique for the YOLO algorithm that eliminated the need for anchor points. This technique separated the detection features from those utilized for recognition to minimize inter-feature interference and augmented detection accuracy. Moreover, an algorithm for enhancing underwater images was based on Retinex image enhancement techniques. Empirical investigations utilizing submerged datasets were performed to authenticate the effectiveness of the implemented optimized YOLO approach. The work in [18] developed a monitoring system for fish farming that utilized approaches for accurately identifying both trajectories and fish count. Initially, the authors improved the quality of indistinct images captured underwater by utilizing Multi-scale Retinex technology. This method provided an enhanced foundation for further image manipulation and analysis. The researchers employed an individual dataset to instruct YOLO in enumerating marine organisms. Integrating the YOLO object identification algorithm with the optical flow technique resulted in an improved approach for tracking fish movements over consecutive video frames, enhancing the accuracy of fish trajectories. The duration of the scheme was considerable.

Chen et al. [19] presented an algorithm for repairing bounding boxes that rely on optimizing the Intersection over the Union (IoU) parameter. The algorithm’s primary focus was optimizing the Mask Scoring R-CNN network, which produced the coarse-grained identification outcomes for the mines. Subsequently, an IoU operation was carried out among the annotated and coarse-grained boxes within the data set to ascertain the optimal correspondences. The optimal correspondence area was employed to rectify the rudimentary boxes. Empirical data indicated that the methodology achieved object detection and localization efficacy in submerged surroundings. However, the precision of identification demonstrated inadequate outcomes. Yan et al. [20] proposed an improved iteration of the YOLOv7 model intended to amplify the accuracy and efficiency of an aquatic target-detecting model in real-time. The study introduced an improved theoretical framework extending the singular stage-target identification model, YOLOv7. This model integrated the CBAM attention mechanism to effectively assign weights and augment the relevant feature data associated with the detection target across spatial and channel dimensions. This strategic approach enabled the more targeted and refined capture of the feature information’s local significance. Moreover, utilizing the SPPFCSPC module in the model served the purpose of minimizing the computational complexity without compromising the model’s perceptual field. Consequently, the speed of inference of the model has been improved.

Mathias et al. [21] presented a study that forwarded a novel technique for detecting submerged objects, specifically emphasizing discerning the foreground entity from the backdrop. The study presented the applications of the Bi-directional Empirical Modes decompositions (BEMD) for generating features from underwater scenes using the blob generation concept. In the work conducted by Sung et al. [22], the application of convolution neural networks in removing and detecting crosstalk noise in images obtained through forwarding scan sonar was investigated. The methodology presented herein detected crosstalk interference by utilizing neural networking techniques and eliminating said interference via a response mechanism informed by this detection results. Jalal et al. [23] described a novel approach to effectively combine the Gaussian mixture and optical flow model into the YOLO-DNN framework, representing a cohesive methodology to identify and classify fish species in uncontrolled underwater video footage.

The authors, Deborah Levy et al. [24], demonstrated the process of marine video recognition and categorization by utilizing an advanced CNN detection system called RetinaNet, coupled with an innovative object tracker known as the Simple Online and Real-time Tracking (SORT) algorithm. Despite utilizing a substantial number of images for its training, the method attained significant levels of precision. The approach exhibited satisfactory performance on datasets obtained from above and below-water environments. The author further disclosed that the efficacy of the RetinaNet object detector, operating at a solitary stage, proved comparable to that of conventional methodologies involving dual stages. The CNN has demonstrated significant advantages in terms of accuracy when compared to alternative methodologies. Nonetheless, notable shortcomings existed in its ability to classify images with varying positions. Moreover, it was essential to note that an extensive training process could be required when a sub-optimal GPU was utilized.

Faster RCNN was improved in the research by Wang and Xiao [25] to detect underwater species like holothurian, scallop, echinus, waterweeds, and starfish in two stages. The Faster RCNN backbone network was upgraded by replacing the VGG-16 structure in the feature extraction module with the Res2Net-101 network to increase all network layers’ expressiveness. The OHEM (Online Hard Examples Mining) technique was created to balance positive and negative bounding box samples. Then, the bounding box regression technique was optimized using GIOU and Soft-NMS. The modified model was trained to utilize the multi-scale training technique to improve reliability, proving that this method was effective in underwater object identification. The YoLoWaternet (YWnet) model proposed by Liu et al. [26] was developed on the YOLOv5 framework for complicated underwater species detection. Initially, a convolutional block attention module (CBAM) improved feature extraction for blurry images, and a novel feature fusion network, the CRFPN, transferred essential information and detected submerged objects. The skip residual C3 module (SRC3), a new feature extraction module, merged data from different scales to reduce data loss during transmission. The decoupled head separated regression and classification algorithms to improve detection, and the EIoU loss function accelerated convergence. Finally, YWnet’s experiments showed outstanding results.

### 2.1. Inference

Numerous studies have indicated that image enhancement procedures could enhance the quality of images. Furthermore, using various derivatives generated from these operations on original images could augment the variety of data in a dataset, thereby boosting its quality and enhancing the performance of models. Table 1 represents the advantages and disadvantages of existing UOD methods. This research supports the adoption of the DL-based BiLSTM-RBM model as a means to enhance the precision of object detection.

**Table 1:** The existing underwater object detection methods using DL with its advantages and disadvantages.

Author	Dataset	Method	Advantage	Disadvantage
Krishnan et al., [13]	URPC 2020, brackish and UOT32	HDCNN-UOD	More layers capture complex image features.	Notwithstanding, the enhancement of learning capacity remains an ongoing challenge, thereby requiring continual improvement.
N. Jiang et al. [14]	unique dataset	YOLOv3	The proposed method has demonstrated greater efficacy in detecting markers across a wide range of environments.	Better DL topology design is still an issue, and no perfect approach exists.
Han et al., [15]	Unique robot-based dataset	deep CNN method	Improved accuracy and speed of detection.	This method does not improve accuracy due to a shortage of underwater samples in the dataset and similar images with identical backgrounds.
L. Chen et al. [16]	URPC2017 and URPC2018	SWIPENet	This method can improve cross-object detection accuracy in complex environments.	The self-attention layer’s computation burden grows exponentially with higher image resolutions.
Wang et al. [17]	URPC dataset	YOLO	This method improves detection accuracy by reducing feature interference.	This method improves detection accuracy by reducing feature interference.
H.E.D Mohamed et al. [18]	dataset containing 400 images of golden fish collected from the internet	YOLO	This method detects fish accurately in poor water.	The processing is time-consuming, and real-time detection is challenging.
Chen et al. [19]	unique dataset	Scoring R-CNN network	This method performs well in detecting and localizing objects underwater.	Underwater images have low detection accuracy due to colour shift and low contrast.
Yan et al., [20]	URPC dataset and Brackish dataset	improved YOLOv7	This method minimizes the loss of feature info and computation.	Despite improvements, YOLOv7-AC still has false and missing detections in complex underwater environments.
Mathias et al. [21]	video sequence	BEND	The integration of colour and motion features serves to enhance the resilience of the proposed method.	High-quality images for detecting targets underwater are a significant challenge.
Sung et al. [22]	Unique dataset	CNN	This enhances processing speed and memory usage.	It is not ideal for complex underwater environments.
Jalal et al. [23]	Two video data sets, i.e., the LifeCLEF 2015 data set from the Fish4Knowledge database	YOLO-DNN	This method is effective for detecting dense objects in underwater environments and can be used for marine object detection.	It is not suitable for underwater complexity and dynamism.
Deborah Levy et al. [24]	Unique dataset	RetinaNet	It is important to propose a marine object-detection framework.	These fast and easy methods produce low accuracy with poor-quality underwater images.

### 2.2. Research gap

The reviewed existing works in UOD primarily focus on employing various DL techniques, such as YOLO variants, Faster RCNN, and CNN, to address challenges related to illumination, noise, and object classification in underwater environments. While these studies have made significant contributions by introducing novel methodologies and achieving commendable results on different datasets, there exists a notable research gap. Specifically, the majority of the reviewed works concentrate on enhancing detection accuracy, often through modifications to existing DL architectures or the introduction of innovative algorithms. However, a crucial aspect that requires further exploration is the development of models that can effectively handle the dynamic and variable nature of underwater environments. The hybrid Bi-LSTM and RBM approach in this research paper is proposed to address this gap by presenting a comprehensive solution that combines the advantages of sequence processing (Bi-LSTM) and hierarchical feature learning (RBM) to improve object detection accuracy in underwater scenarios.

## 3. Hybrid Bi-LSTM and RBM approach

**Fig 1:**
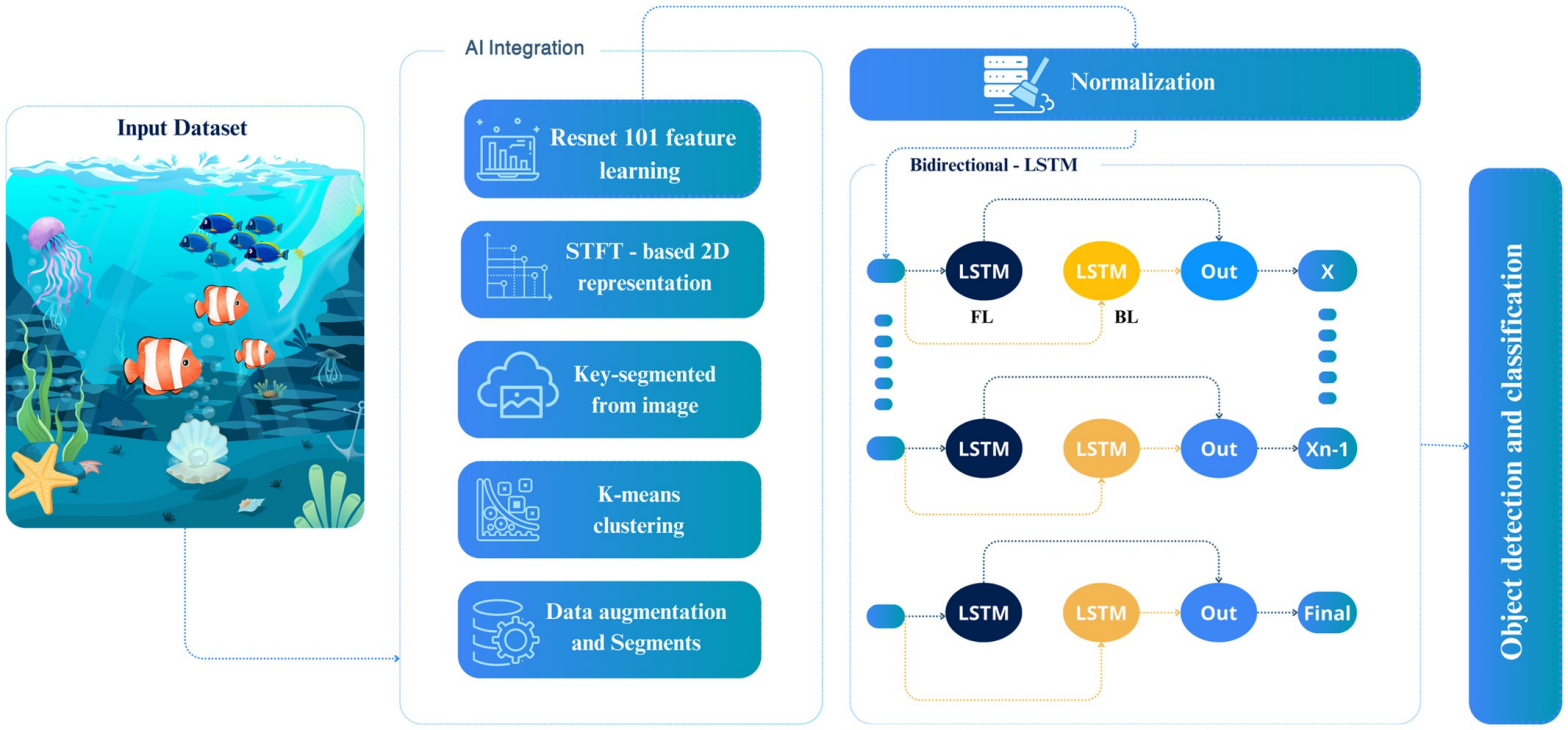
Block diagram of Bi-LSTM-RBM based UOD technique.

The present research has developed an innovative Bi-LSTM-RBM framework, which exhibits a remarkable ability to identify and monitor objects in underwater effectively. The Bi-LSTM-RBM approach encompasses a sequence of procedures, including converting videos into frames, data augmentation, delineating key segments through clustering, feature extraction based on Resnet 101, prediction based on Bi-LSTM-RBM, and classification. Upon successfully identifying the objects, they undergo a subsequent conversion process to be reinstated as tracked video. Fig 1 depicts the block structure of the UOD approach using Bi-LSTM-RBM. The video undergoes augmentation and is subsequently segmented into multiple temporal sections, wherein the variance between sequential segments is determined. Subsequently, the disparity metric is employed to quantify similarity, and the optimal value for K is determined by applying the shot boundary detection technique for clustering. Subsequently, a key segment near the cluster’s centre is selected from each group or sequence and employed for similarity determination via the employment of RBF. The methodology extracts discriminative and salient features from object spectrography through a transfer learning approach within the second primary module. More specifically, the feature learning process is contributed. The acquired characteristics are subjected to normalization to enhance the performances utilizing the standard deviation and mean. In the preceding phase, the extracted normalized CNN characteristics are utilized as input to the proposed deep BiLSTM-RBM to acquire knowledge of temporal patterns and identify the sequential data within the sequence while analyzing subaquatic entities.

### 3.1. Pre-processing

Traditionally, data pre-processing was conducted before data augmentation techniques were implemented. The visual representations underwent pre-processing measures, including, but not limited to, resizing, orientation adjustment, and colour correction. Using pre-processing techniques accelerates the procedure of model inference and effectively mitigates the duration of model training. If the input images have a significantly large size, reducing their size will considerably decrease the training time of the model while still maintaining its performance. They are employed on both practice and testing sets. Image augmentation was solely implemented on the training dataset, where the augmentation generates novel training instances from the pre-existing training instances. "To augment the training, a data augmentation process was implemented." Data augmentation approaches were initially applied to mitigate overfitting and expand the data set size. This study utilizes data augmentation techniques, including rotation, translation, and flipping, to substantially expand the total images contained within the training dataset. Furthermore, input images undergo rotation at disparate angles in rotation-based data augmentation, namely 90◦, 180◦, and 270◦. The input images undergo a flipping process whereby they are mirrored both horizontally and vertically.

Following data augmentation, several observations were conducted on various frame durations to effectively determine the optimal window size of 500ms for segmenting a single into multiple segments. A singular label was apportioned to all the segments within the observations and subsequently inputted into the K-mean clustering algorithm [27] to group similar segments. The K-mean clustering algorithm is a highly prevalent technique for segregating large datasets [28]. The conventional approach in the K-mean clustering technique for determining dissimilarities among elements involves the utilization of the Euclidean distance matrix [29, 30]. In this study, the RBF was utilized as a replacement for the Euclidean distance matrix in the K-means algorithm to calculate the dissimilarity measure between two frames. The Radial Basis Function (RBF) methodology has been employed as a non-linear technique, resembling the functioning of the human cerebral cortex, for computing variances and identifying patterns. The proper consideration of the "K" value to partition data into "K" clusters is an equally crucial aspect to be considered.

The K-means utilizes a random initialization approach to determine the appropriate variable `K’ values. However, a more dynamic approach is achieved through the shot boundary detection method, enabling the choice of the optimal `K’ values for individual files. This method estimates similarity and has been documented in [31]. The calculation of the pairwise difference occurs within sequential frames, whereby an increase in value exceeding the predetermined threshold results in the incremental addition of one unit to the variable denoted as "K." Upon applying the K-mean algorithm for segment clustering, one key segment is chosen from each cluster that closely approximates the cluster centroid, as determined through the utilization of the RBF distance approach outlined in the subsequent sections. The identified crucial elements are transformed into spectrograms utilizing the STFT approach to facilitate their two-dimensional representations.

### 3.2. RBF-based similarity measure

The use of RBM helps the model capture meaningful latent features during pre-training, which aids in the initialization process. This improves convergence during the training of deep neural networks and prevents the model from being trapped in poor local minima, which is a concern when dealing with such noisy and complex data. In the present segment, a comprehensive account of the measure of non-linear similarity is employed for the analysis of video segments. The radial basis functions (RBFs)-based similarity approach for video image processing is investigated. The RBF employs non-linear computation techniques to determine the similarity among segments by employing the principle of non-linearity, as described in reference [32]. The human brain’s visual perception component operates based on a non-linear process to discern and identify various segments. Consequently, this method is utilized in the developed model to determine the similarity measure across audio segments.

The RBFs are utilized to emulate the non-linear human perception method to effectively capture and calculate the level of resemblance within various audio segments. The method exhibits non-linearity and is founded on radial basis function networks, as established in reference [33]. A mapping function is employed to determine the similarity between audio segments. The regularization technique has been employed to determine the mapping function of the fundamental RBF. The present model utilizes the Radial Quadratic (QR) kernel with 1D Gaussian distribution [34], which substantially satisfies a crucial prerequisite of the regularization technique. Specifically, it facilitates smoothing the mapping function given by Eq (1).


$$\Phi_{\text{RQR}}\left(x\right)=\text{exp}\left(\frac{{\left(x-c\right)}^{2}}{2\sigma^{2}}\right)*\sigma^{2}{\left(1+\frac{{\left(x-c\prime \right)}^{2}}{2\alpha }\right)}^{-\alpha }$$ (1)

The parameters *c* and *σ* serve to denote the centre and width of the function. The transformation of the Gaussian involves the use of Φ(c) as a means of determining the degree of similarity and distance between the input variable, denoted as ’*x*’, and the centre parameter, ’*c*’. The generation of various RBFs is facilitated by an RBF network (RBFN) that possesses an exceptional aptitude for non-linear approximations [35]. The RBFN operates through the derivation of the function *f*(*x*) using RBFs, as indicated below in Eq (2).


$$f\left(\bar{x}\right)=\overset{N}{\underset{i=1}{\sum }}\;w_{i}\Phi_{\text{RQR}}\left(x,\sigma_{i}\right)$$ (2)

The mapping function’s expanded form is given in Eq 3:

$$f\left(\bar{x}\right)=\overset{N}{\underset{i=1}{\sum }}\;w_{i}\;\text{exp}\;\left(-\frac{1}{2\sigma_{i}^{2}}\sum_{j=1}^{P}{\left(x_{j}-\sigma_{ij}\right)}^{2}\right)$$ (3)

The present study utilizes the basis function denoted as *P*, which comprises a collection of $\left\{\Phi_{{\text{RQR}}_{1}}\left(\sigma_{1}\right),\;\Phi_{{\text{RQR}}_{1}}\left(\sigma_{1}\right),‥,\Phi_{{\text{RQR}}_{\text{P}}}\left(\sigma_{P}\right)\right\}$. The parameter $\Phi_{\text{RQR}}\left(\bar{\text{x}},{\text{c}}_{\text{i}}\right)$ signifies the width of the function, while the index "*i*" denotes its centre. Meanwhile, the mapping functionality *f*(*x*) was described as the summation of "N" Gaussian components. To mitigate the computational burden associated with the network, the one-dimensional Gaussian RBF is employed for each segment of the object.


$$f\left(\bar{x}\right)=\overset{P}{\underset{i=1}{\sum }}\;w_{i}\;\Phi_{\text{RQR}}\left(x_{i},\sigma_{i}\right)$$ (4)

$$f\left(\bar{x}\right)=\overset{P}{\underset{i=1}{\sum }}\;\text{exp}\left(-\frac{{\left(x_{i}-c_{i}\right)}^{2}}{2\sigma_{i}^{2}}\right)$$ (5)

In Eqs (4) and (5), $\bar{x}$ refers to a specific component of an utterance object, while *c* denotes the central point of the RBFs. The particular object segment’s width of the RBF was represented by *σ*_*i*_, where *i* ranges from 1 to *Q*. Eq (5) is utilized to determine the level of similarity between two video segments, which is then characterized by the variable width of all the RBFs to produce the value "*Q*". The adjustment of parameters, incorporating non-linear weights, and estimation of sample variances in relation to the relevance sets are acquired through a comprehensive approach, as shown in Eqs 6 and 7.


$$\sigma_{i}=\text{exp}\left(\alpha ,Ob_{i}\right)$$ (6)

$$Ob_{i}=\sqrt{\left(\frac{1}{Q-1}\overset{Q}{\underset{j=1}{\sum }}{\left(x_{ji}-{\bar{x}}_{i}\right)}^{2}\right)}$$ (7)

If a particular segment of the object holds higher significance, it is anticipated that the associated standard deviation value amongst the segments of the object will be small. A high standard deviation value indicates the segments of the object to be irrelevant. Consequently, the alterations in distance are regarded as very sensitive for the segments that possess a smaller term designated as "σ".

### 3.3. Feature extraction

The feature extraction sub-section presents an intricate analysis of the process of feature extraction and Recurrent Neural Network (RNN) in the context of recognizing objects from underwater videos, which entails sequential data processing. CNN remains the preeminent source for detecting and recognizing latent information within contemporary data. In contrast, the videos are transformed into several segments, with each segment being represented by CNN features. Subsequently, a deep Bi-LSTM was implemented to extract sequential information from the features. The visual data captured by video recordings often contains redundant data that necessitates significant computational resources and detrimentally affects the efficiency of the utilized model. According to this imposed limitation, an innovative approach was introduced for identifying the most prominent sequence within an observation using K-mean and RBF. The chosen sequences for all the segments are transformed into spectrograms, which depict the frequency distribution over time in a two-dimensional (2-D) format by applying the STFT algorithm.

**Fig 2:**
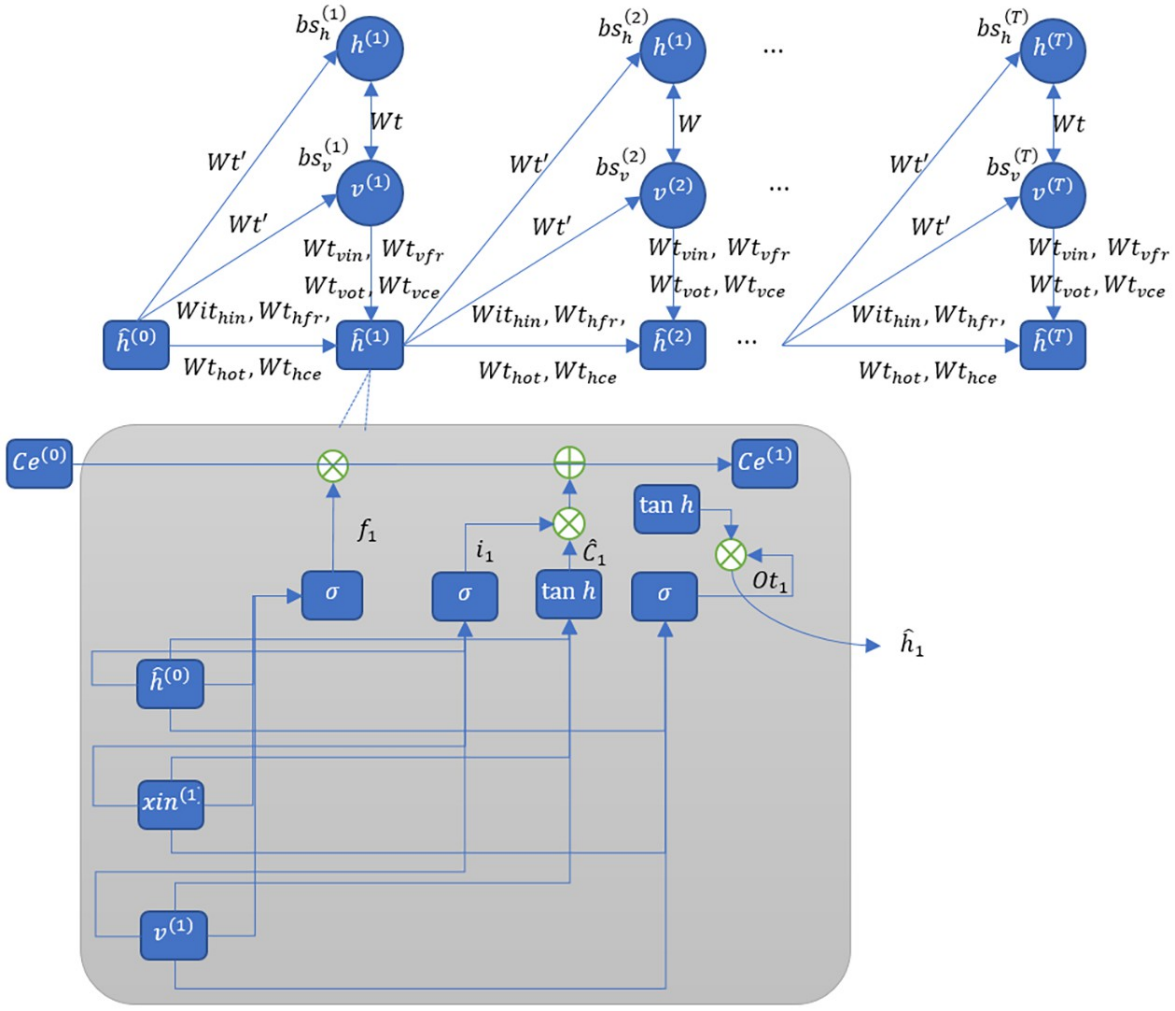
Architecture of LSTM-RBM.

The present methodology involves employing a pre-existing CNN model, specifically the Resnet101 model, with its pre-trained parameters, to extract higher-level distinguishing characteristics from a sequence of spectrograms [36]. This is accomplished through a transfer learning model, which employs the final layer designated as "FC-1000" from the Resnet101 model. The RBM was utilized to extract the salient features of sequence information, but it cannot capture the dynamic temporal patterns that emerge between successive sequences. Unlike RBM, the RNN-RBM method demonstrates superior abilities in extracting temporal features from sequences. However, it must maintain the inadequacies of RNNs, namely the vanishing gradient and the incapacity to grasp sequences’ long-term dependencies [37]. Thus, to exceed this limitation, LSTM-RBM was employed. The LSTM-RBM, a derivative of the RNN-RBM, exhibits a structure similar to that of the latter, as depicted in Fig 2. To incorporate the temporal features of sequences, the LSTM hidden layer’s output is externally linked to the RBM’s hidden and visible layers. The present study utilizes the temporal data accumulated through the LSTM method to facilitate the modification of RBM’s two biases, namely, $bs_{h}^{\left(t\right)}$ and $bs_{v}^{\left(t\right)}$. The update process follows the methodology articulated in Eq (4). Divergent strategies exist between the underlying mechanisms of RNN and LSTM [38, 39]. As illustrated in Fig 2, it presents the internal architecture of LSTM. The RBM’s present output of the visible layers (*v*(*t*)), the LSTM’s present input (*cx*(*t*)), and the LSTM’s hidden layer output at the prior time moment (*ch*(*t*)) have a direct impact on the present output of the LSTM. Henceforth, the updated equation for the internal input gate (*in*_*t*_), output gate (*ot*_*t*_), forget gate (*fr*_*t*_), and output states (*Ce*_*t*_) of the Cells in the LSTM models were formulated as follows in Eqs 8 to 11.


$$in_{t}=g\left(Wt_{xin}x^{\left(t\right)}+Wt_{hin}h^{\left(t-1\right)}+Wt_{vin}vi^{\left(t\right)}+bs_{in}\right)$$ (8)

$$fr_{t}=g\left(Wt_{xfr}x^{\left(t\right)}+Wt_{hfr}h^{\left(t-1\right)}+Wt_{vfr}vi^{\left(t\right)}+bs_{fr}\right)$$ (9)

$$ot_{t}=g\left(Wt_{xot}x^{\left(t\right)}+Wt_{hot}h^{\left(t-1\right)}+Wt_{vot}vi^{\left(t\right)}+bs_{ot}\right)$$ (10)

$$Ce_{t}=fr_{t}*Ce_{t-1}+it_{t}*\text{tanh}\left(Wt_{xce}x^{\left(t\right)}+Wt_{hce}h^{\left(t-1\right)}+Wt_{vce}vi^{\left(t\right)}+bs_{ce}\right)$$ (11)

The weight matrices are denoted as *Wt*_*xin*_, *Wt*_*xfr*_, *Wt*_*xot*_ and *Wt*_*xce*_, correspond to the connections between the input and the three gates, namely the Forget Gate, the Output Gate, and the Cell Gate, as well as the Cell itself within the LSTM model at present. The weight matrix associated with the hidden layer output during the prior time step was denoted by *Wt*_*hin*_, *Wt*_*hfr*_, *Wt*_*hot*_ and *Wt*_*hce*_. On the other hand, *Wt*_*vin*_, *Wt*_*vfr*_, *Wt*_*vot*_ and *Wt*_*vce*_, the weight matrix corresponding to the reconstructed visible layer’s RBM output, and its related biases were given by *bs*_*in*_, *bs*_*fr*_, *bs*_*ot*_ *and bs*_*ce*_. The sigmoid function is denoted by the symbol "*g*." At this point, the computation of the LSTM output, *chi*_*t*_, in Eq 12 can be ascertained through the subsequent algorithm:

$$chi_{t}=op_{t}*\text{tanh}\left(Ce_{t}\right)$$ (12)

Additionally, PCD and Gibbs sampling were further implemented to accelerate the sampling efficiency in the RBM.

### 3.4. LSTM-RBM with fine-tuning

This model was proposed to extract salient features from speed sequences characterized by their rapid updating and sourced from multiple origins. In the context of a singular model, the abstracted characteristics are confined in their scope, thereby posing a challenge in fine-tuning the parameters for multiple models. As such, it is proposed to employ the LSTM-RBM approach with fine-tuning technique to predict objects in underwater, utilizing the concept of transfer learning as elucidated in the literature. One plausible approach is to derive a set of generalizable characteristics from the feature extraction process of the pre-training model employing LSTM-RBM methodology. Alternatively, it is possible to enhance the initial parameter values and constrain selected parameters to the initial values to mitigate training expenses. Transfer learning is a prevalent technique in ML whereby an established model is adapted to a new domain through minor modifications. The method is primarily segregated into two distinct categories. There are two approaches to feature extraction: the first involves using all layers, except the fully connected layer on the top, to extract features that are subsequently predicted or classified by various ML techniques; the second is fine-tuning. The selection of fine-tuning layers, whether complete or partial, is contingent upon prevailing conditions and is exercised with unimpeded discretion. Given that the properties deduced from the fundamental model are ubiquitous across the input data, with distinctiveness being discerned from the models close to the uppermost layer, the conventional method optimizes the fundamental models through fine-tuning.

**Fig 3:**
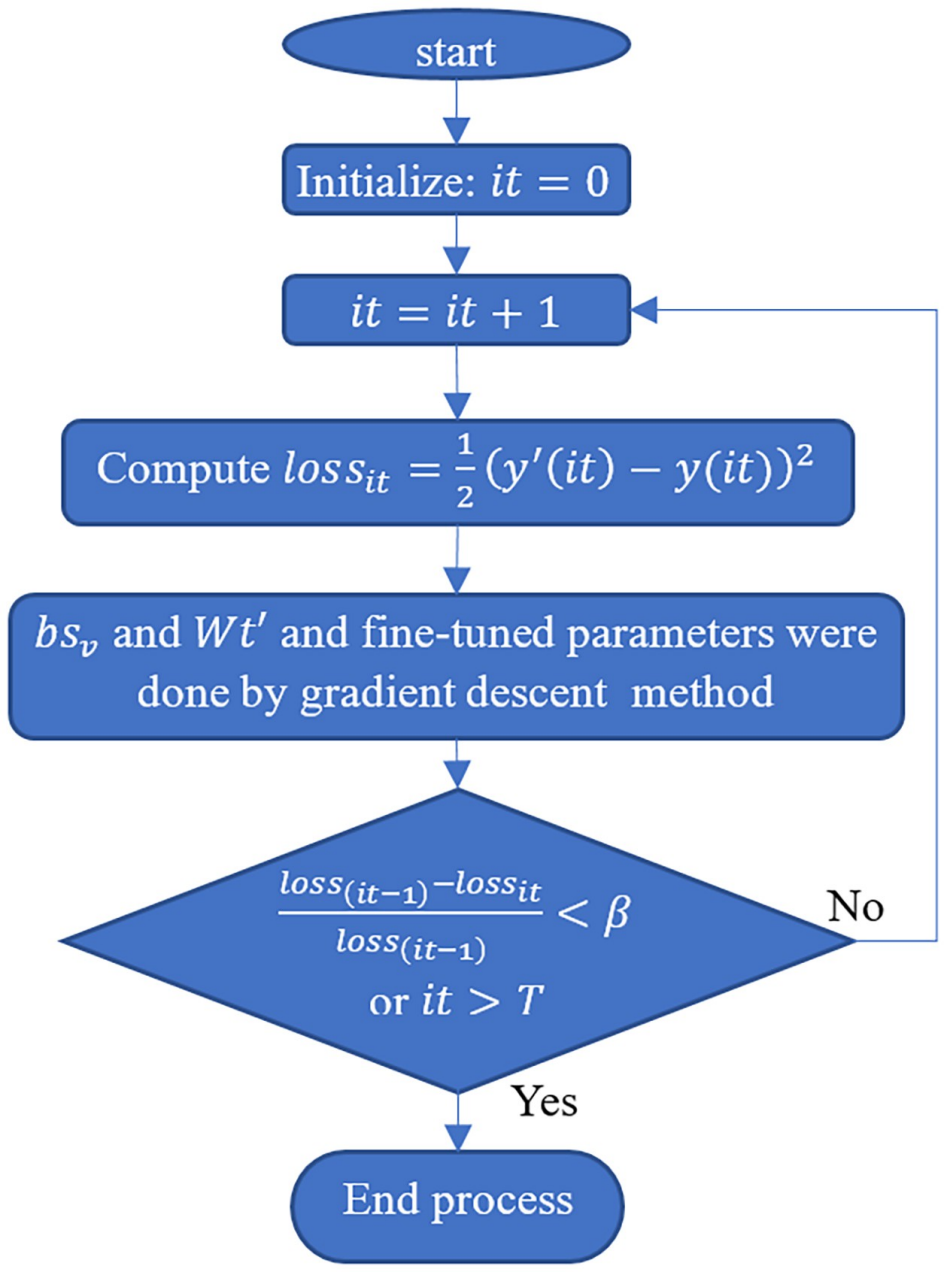
LSTM parameters are updated in the fine-tuning process using parameters updated BP algorithm.

Henceforth, this research opted to fine-tune the LSTM-RBM approach to enhance the expeditious expression of the speed sequence. The LSTM-RBM algorithm presented herein is founded on a layer-by-layer greedy learning approach. Through the process of fine-tuning, its parameters have been properly initialized, thereby requiring only a selective fine-tuning of parameters for further optimization. The Back Propagation (BP) algorithm was predominantly employed in the research to update the LSTM-RBM parameters through fine-tuning. This serves as the learning approach for the parameters. The procedural instructions for training are exemplified diagrammatically through a flow chart, which can be found in Fig 3. In this context, $y_{\left(it\right)}^{\prime }$ has been identified as the predicted value. At the same time, "*y*_(*it*)_" represents the actual value. Additionally, the threshold parameter β has been incorporated to facilitate parameter updates and avoid overfitting. It should be noted that the total number of iterations in this particular instance is denoted by the variable "T". When the value of beta exceeds an appropriate threshold, a prolonged duration will be needed to obtain superior parameters for the model. Conversely, if the beta is set below an appropriate threshold, the error curve will oscillate, thereby hindering model convergence. As emphasized in previous research [8], the initial optimal value of beta was 0.01. Therefore, for the study, beta is likewise established at 0.01.

### 3.5. Bi-directional LSTM

The output of the BiLSTM framework is influenced by both antecedent and subsequent segments of the sequence rather than just relying on a single segment at any given time step [40]. This study employs the multi-layered concept of the LSTM networks. Specifically, a two-layer network is utilized for both the proposed method’s forward and backward passes. The schematic depiction of the proposed multi-layer BiLSTM is illustrated in Fig 3. The provided graphic depicts the training phase of the bidirectional RNN, which incorporates the hidden state from both the forward and backward passes in the output layers, thus showcasing the external structure. Upon completion of the output layer, the process of evaluating the cost and undertaking validation is undertaken. This is followed by the backpropagation approach, which facilitates the systematic adjustment of weights and biases. The network’s validation process involves utilizing 20% of the data, distinct from the training data, to assess the credibility of the network’s predictions. This evaluation is conducted by computing the error rates in the validation instances employing the Cross-Entropy method. The approach of Adam optimization [41] is utilized to minimize the cost through a learning rate of 0.001. The structure of the Bi-LSTM network is characterized by the presence of deep cells governing the forward and backward pass. This results in an enhanced network capability to compute outputs from preceding and succeeding sequences over time, owing to the bidirectional nature of its processing.

## 4. Results and discussion

**Fig 4:**
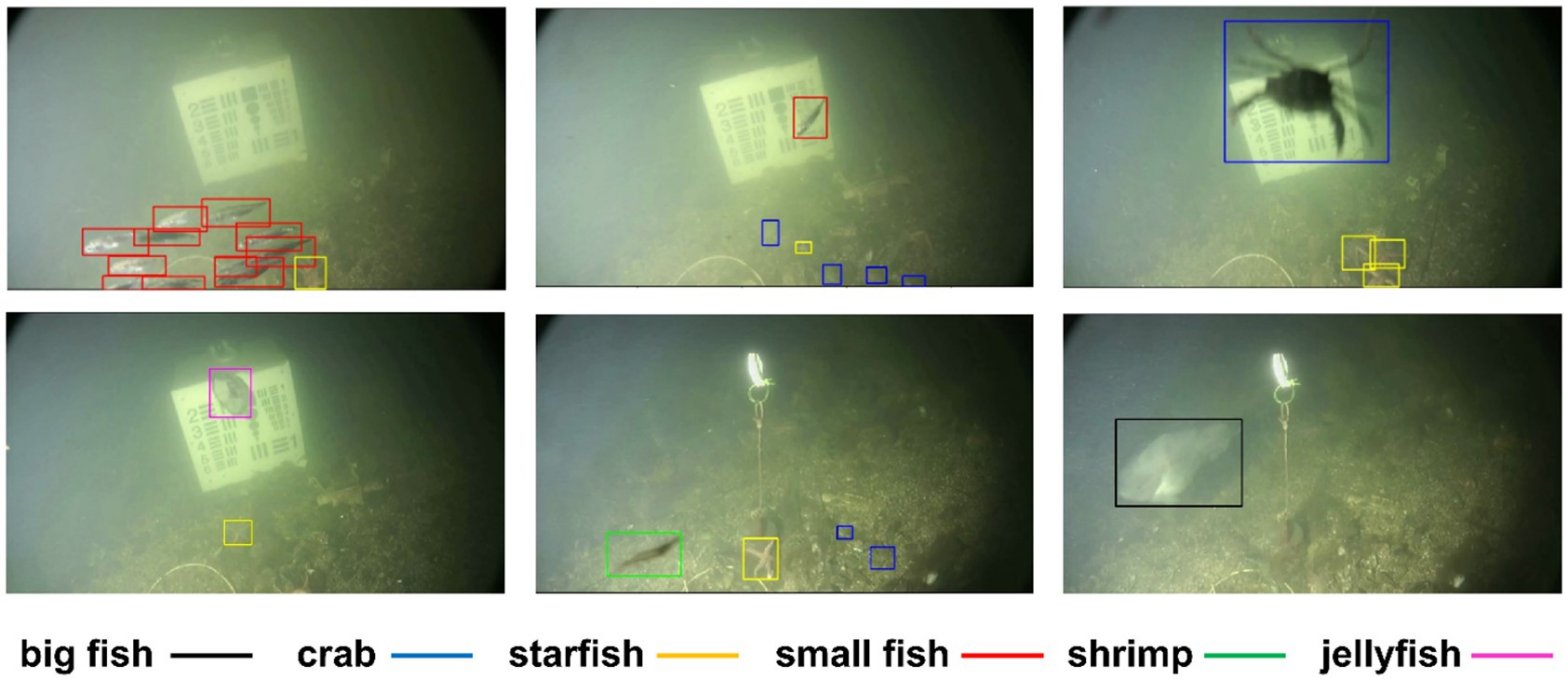
Sample images from brackish dataset [42].

The section 4 results and discussion section present an evaluation of the performance of the Bi-LSTM-RBM-based UOD model proposed herein, measured in terms of APE, ASR, and AFPSS accuracy, and subsequently compared against prevailing UOD schemes like HDCNN-UOD [13] and T-YOLO v4 [13]. The dedicated server for underwater object detection (UOD) is equipped with high-performance hardware, including an Intel Xeon processor with at least 8 cores, 32 GB or more of RAM, a 1 TB SSD for fast read/write speeds, a 2 TB HDD for data storage, and an NVIDIA GeForce RTX 3080 with at least 10 GB of VRAM for efficient deep learning model training. It runs on a Linux distribution, such as Ubuntu 20.04 LTS with a software environment that includes deep learning frameworks TensorFlow and OpenCV for image processing. The BI-LSTM-RBM technique underwent performance validation conducted via the use of two data sets, specifically, the brackish [42], and URPC 2020 [43, 44]. Fig 4 depicts the sample image from brackish dataset.

**Fig 5:**
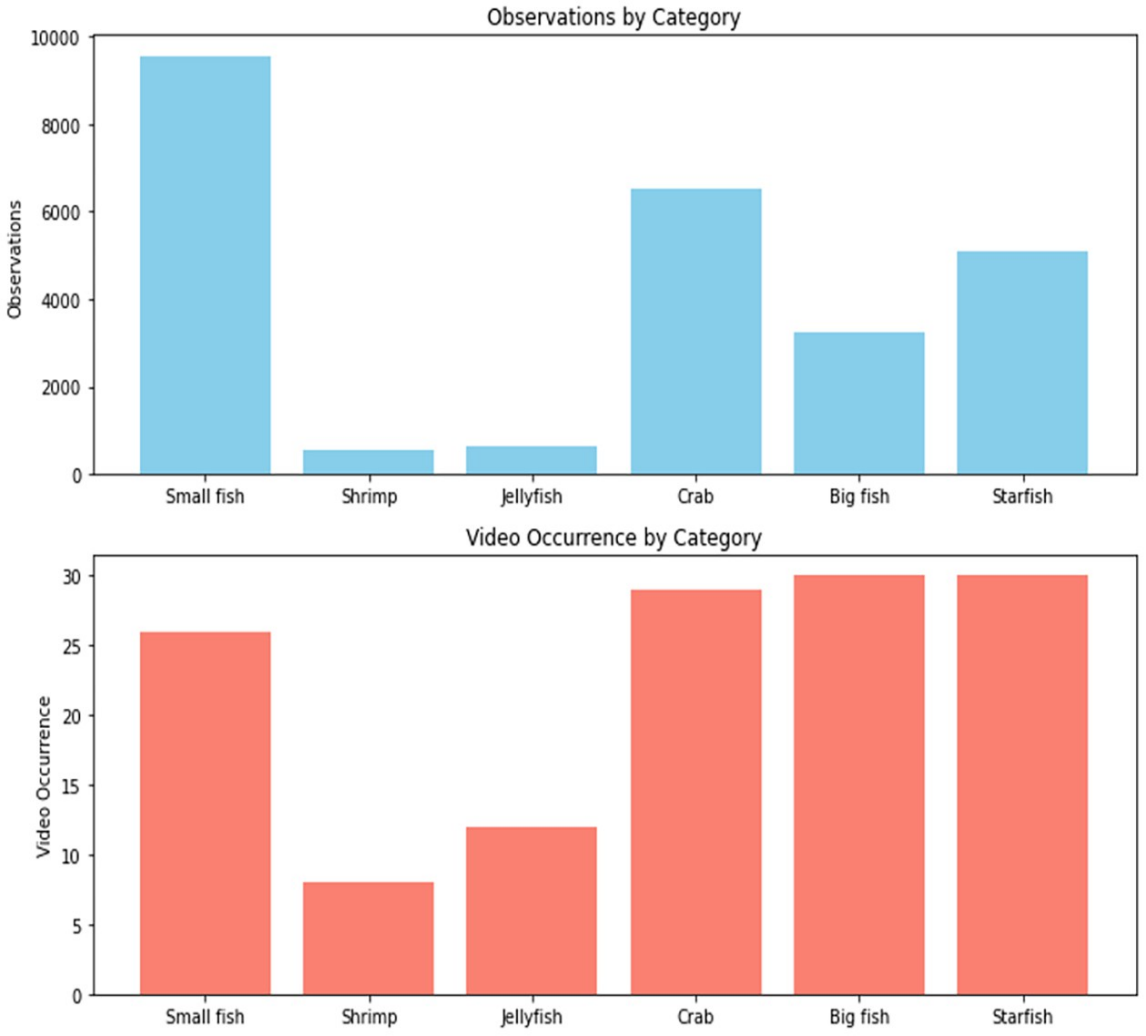
Video occurrences and observation by category of brackish dataset.

**Fig 6:**
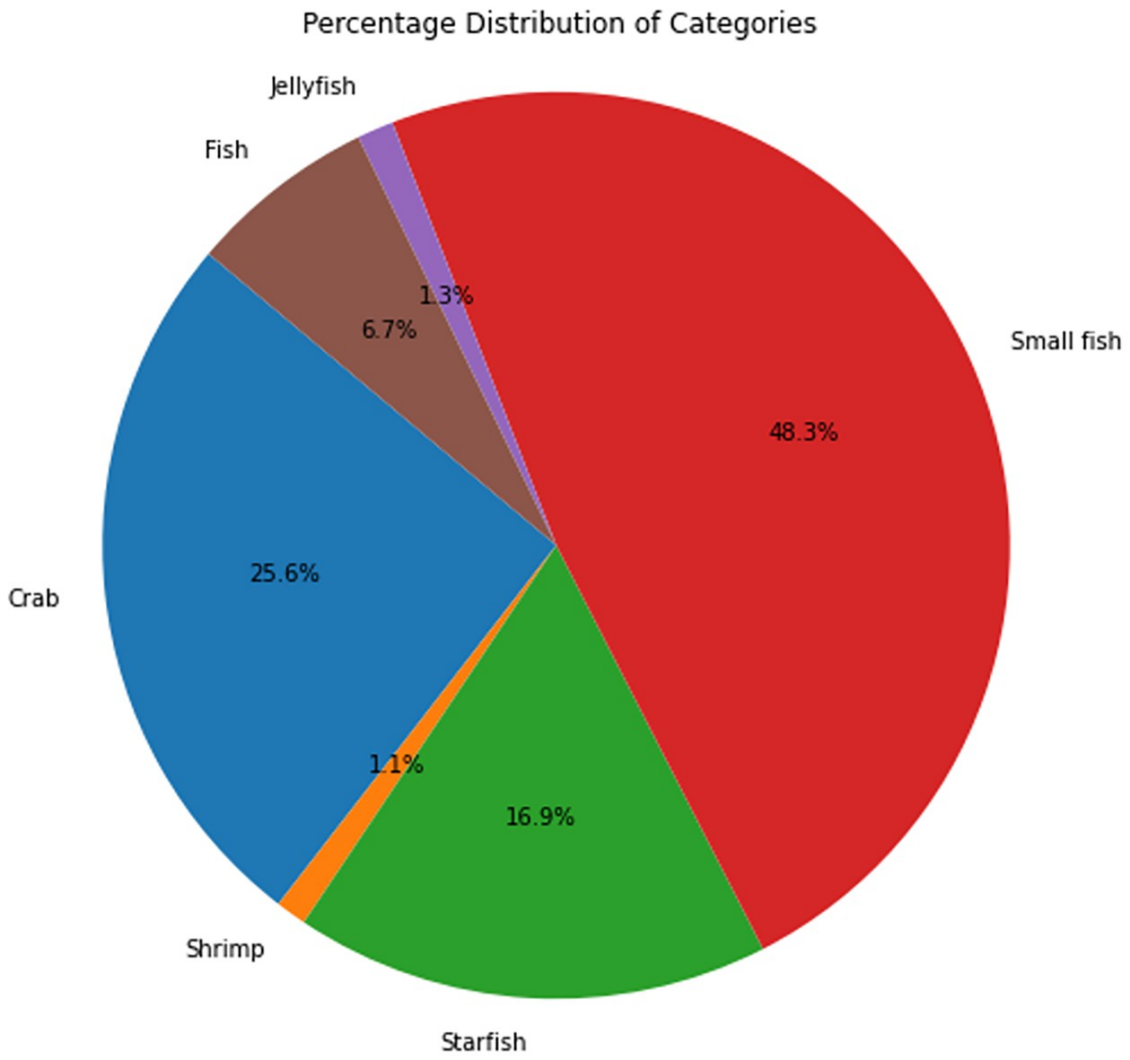
Class distribution of the brackish dataset.

Fig 5 illustrates the occurrences and observations of the brackish dataset categorized by species. Fig 6: illustrates the class distribution of the brackish dataset.

**Fig 7:**
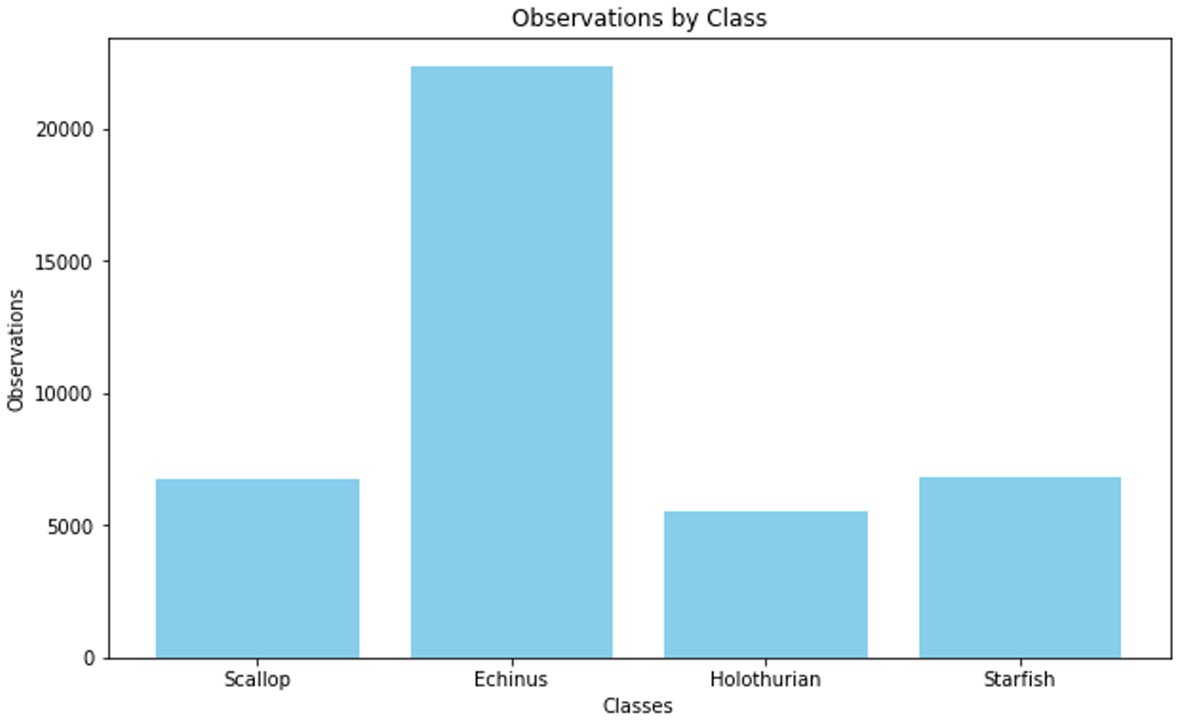
URPC2020 Dataset observation by class.

Fig 7 shows the URPC2020 Dataset observation by class. The findings have revealed that the models have acquired a relatively acceptable average percentage error (APE) of 60.20%, 51.23%, 47.20%, and 40.50%, in sequential order. The BI-LSTM-RBM model has attained a superior achievement in terms of maximum APE, with a percentage of 60.2% compared to alternative models. The proposed model, which utilizes LSTM with RBM and optimized parameters, has successfully reduced its computational complexity and demonstrated superior performance in achieving high APE compared to alternative models.

Table 2 displays the outcomes obtained from the BI-LSTM-RBM-based UOD model for object detection in comparison to other current techniques on the brackish and URPC2020 dataset. The BI-LSTM-RBM-based UOD model exhibited a noteworthy performance in identifying objects denominated as ’Big Fish,’ demonstrating high accuracy (98.5%). By comparison, other models such as HDCNN-UOD, T-YOLO v4, and YOLO appeared to achieve marginally lower levels of accuracy, i.e., 97.94%, 89.41%, and 85%. Simultaneously, the BI-LSTM-RBM-based UOD model has demonstrated a heightened precision in identifying ’Crab,’ with an accuracy of 96.85%. Conversely, the HDCNN-UOD, T-YOLO v4, and YOLO models have decreased accuracy at 90.88%, 87.15%, and 82.74%. The data indicated that the BI-LSTM-RBM-based UOD model achieved superior results in object detection compared to other approaches.

**Table 2:** Accuracy for Brackish and URPC2020 dataset.

Classes	Proposed Bi-LSTM-RBM	HDCNN-UOD	T-YOLO v4	YOLO
Jellyfish	98	89	88	82
Shrimp	98.2	90	90	83
Big fish	98.5	97.84	89.41	85
Crab	96.85	90.88	87.15	82.74
Small fish	98.5	91	88	84
Starfish	97	89	87	85
Holothurian	96	88	88	83
Echinus	97	89	86	84
Starfish	98	90	86	83
Scallop	96	90.05	88	82

**Fig 8:**
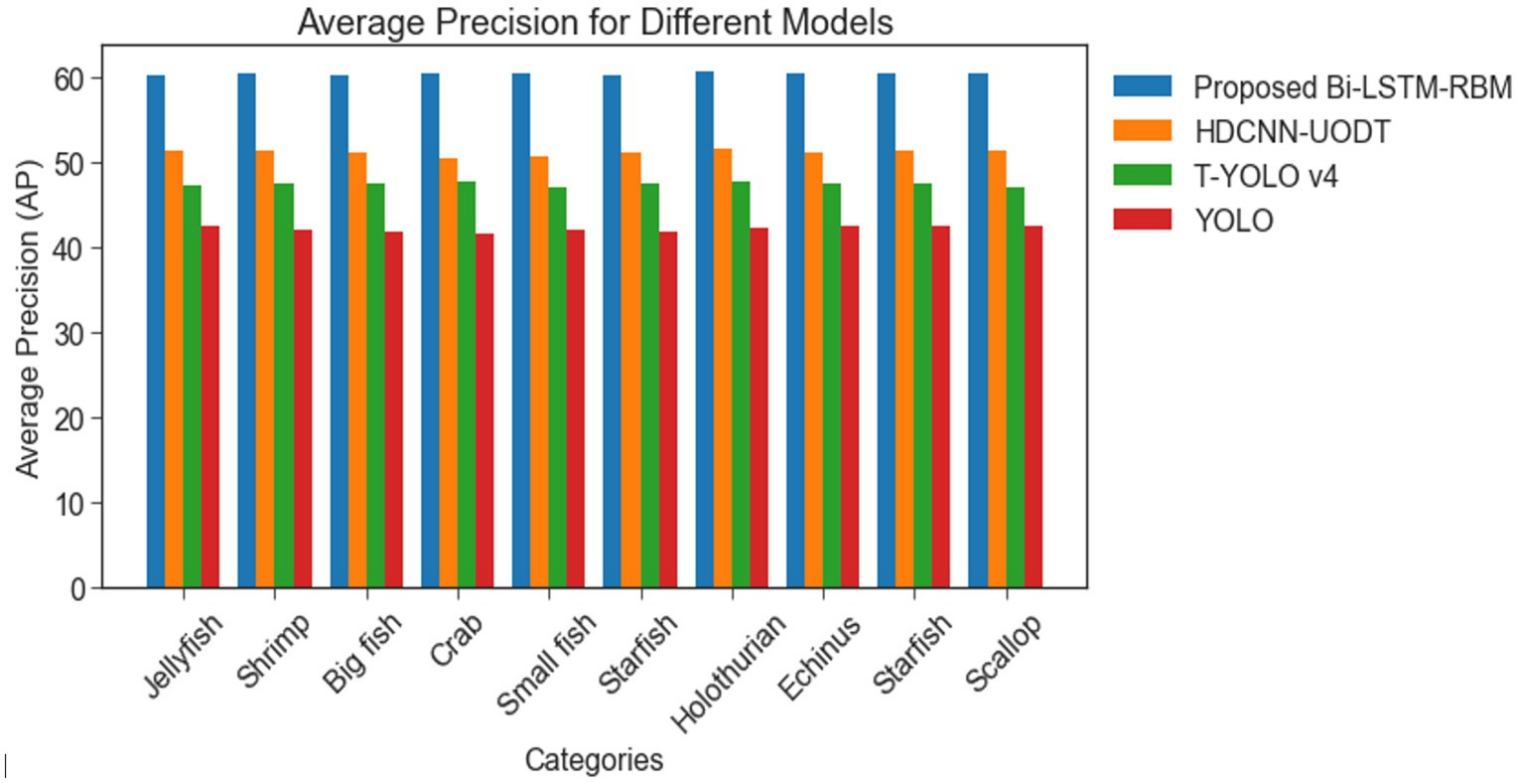
APE performance analysis among Brackish and URPC 2020 datasets.

**Fig 9:**
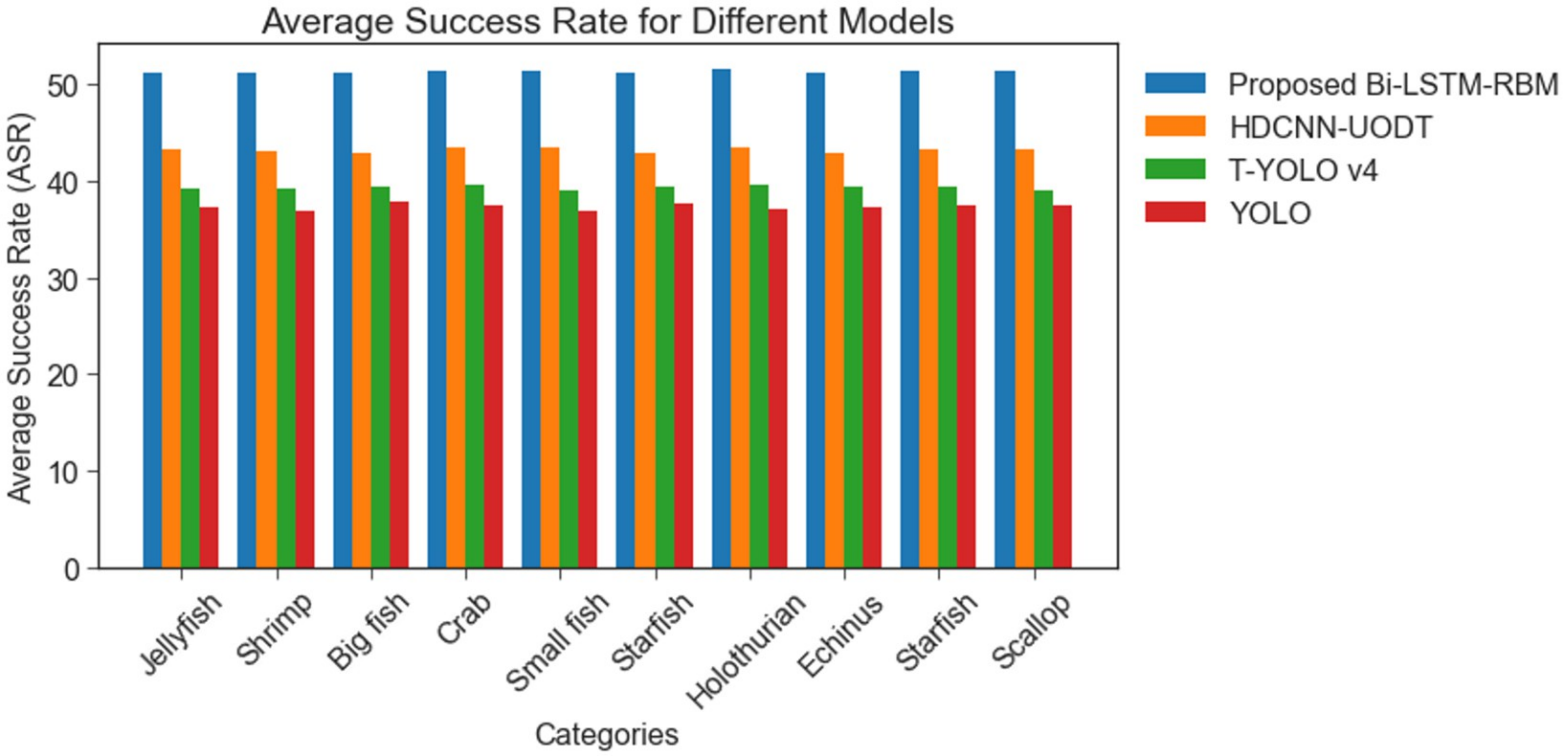
ASR performance analysis among Brackish and URPC 2020 datasets.

Fig 8 shows an evaluation of the average precision (APE) of the BI-LSTM-RBM-based UOD model and other related techniques, namely HDCNN-UOD, T-YOLO v4, and YOLO, applied to the Brackish and URPC 2020 datasets. Fig 9 compares the performance of the BI-LSTM-RBM-based UOD system with that of other techniques, including HDCNN-UOD, T-YOLO v4, and YOLO, on the Brackish and URPC 2020 datasets by analysing their average success rates (ASR) in the examination. The findings suggest that the models have achieved a remarkable level of ASR with 43.19%, 39.70%, and 37.30%. Nonetheless, the BI-LSTM-RBM model has achieved the highest ASR performance of 51.3% when compared to alternative models. The proposed technique attained a high ASR rate owing to its reduced time consumption.

**Fig 10:**
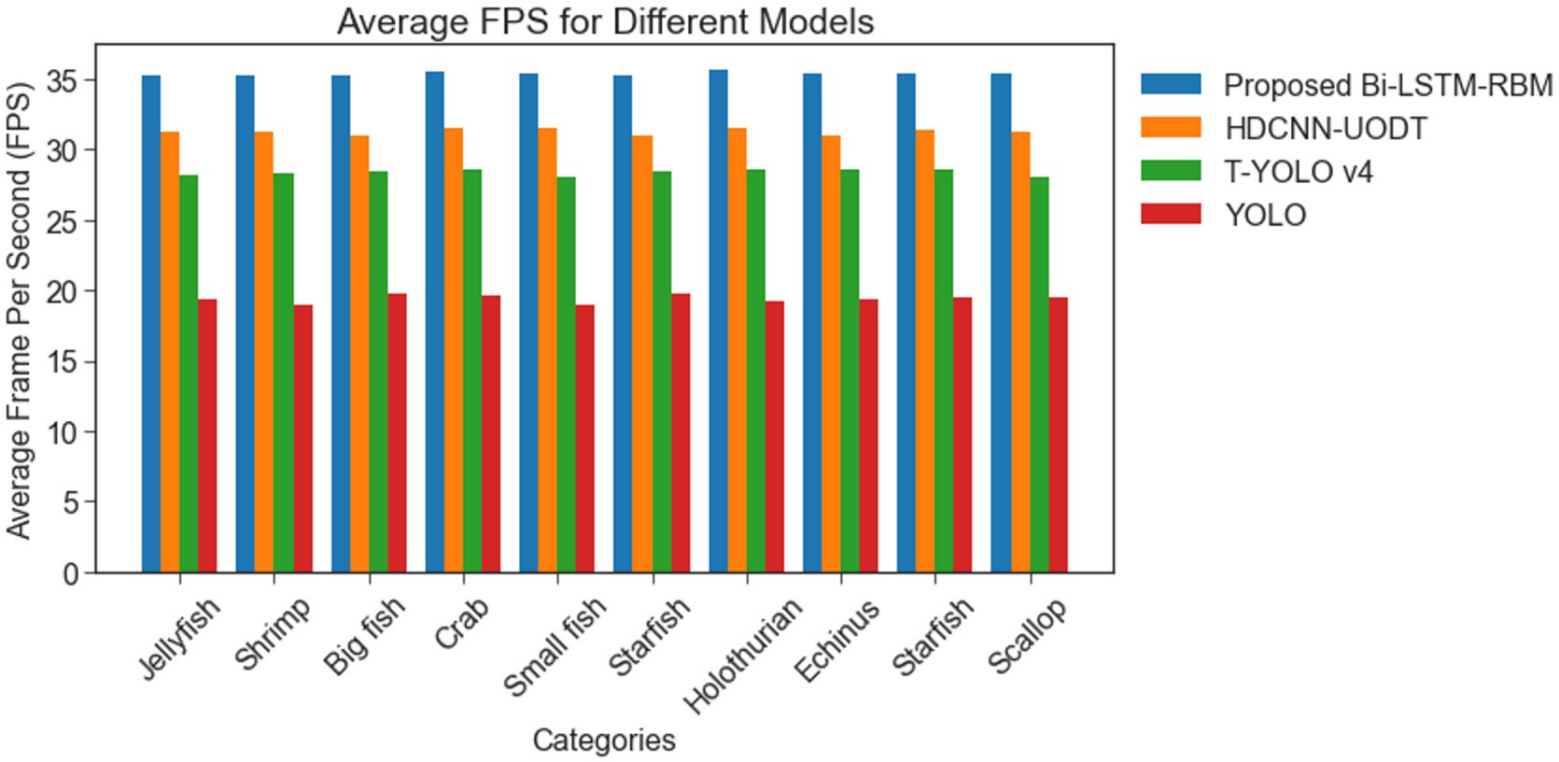
AFPS performance analysis among Brackish and URPC 2020 datasets.

**Fig 11:**
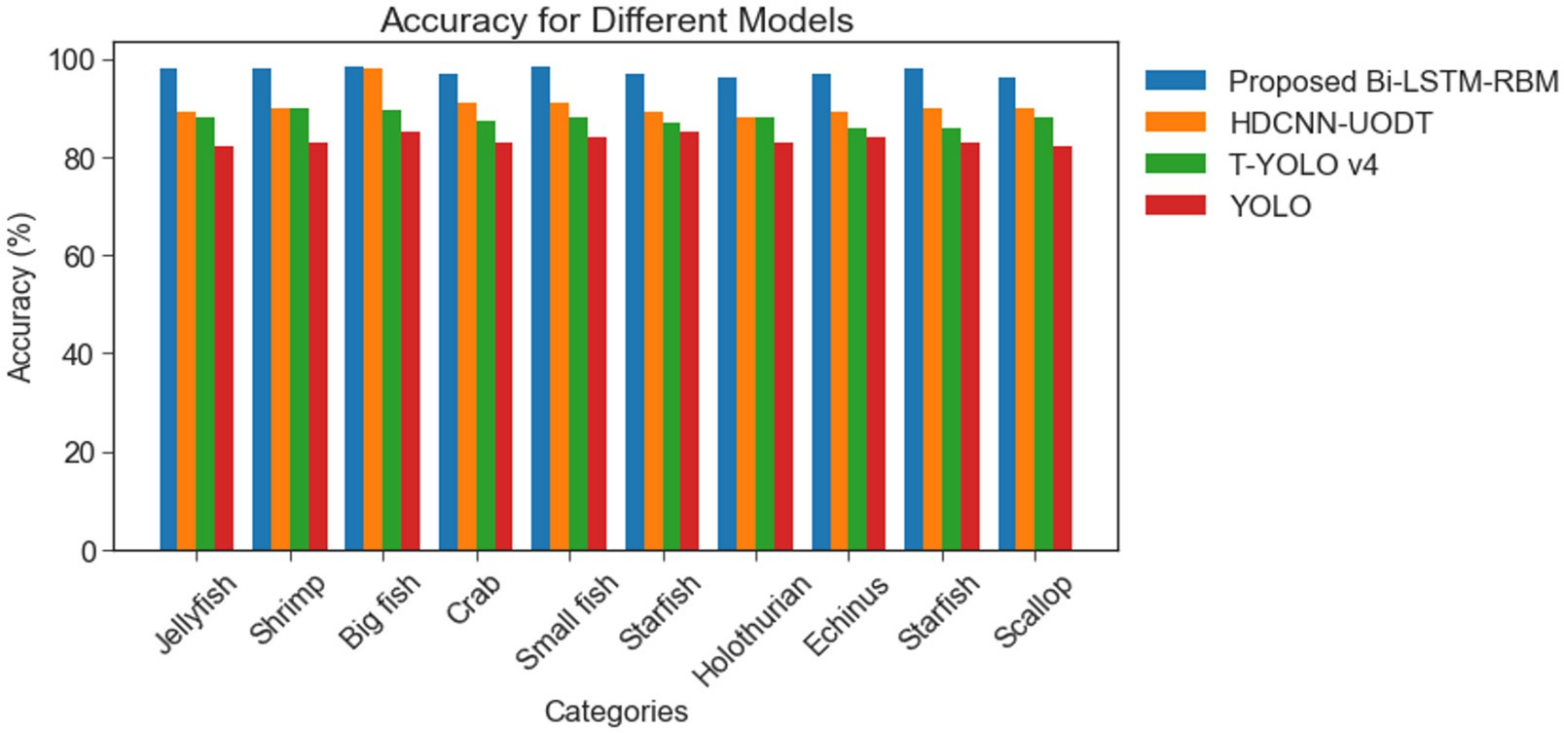
Accuracy performance analysis for Brackish and URPC 2020 dataset.

Fig 10 presents an analysis of the average frame per second (AFPS) of the BI-LSTM-RBM model in comparison to various methodologies, namely HDCNN-UOD, T-YOLO v4, and YOLO, for both the Brackish and URPC 2020 datasets. The findings of the study revealed that the models had achieved AFPS of 31.23%, 28.29% and 19.22%. The BI-LSTM-RBM model has demonstrated superior performance, achieving a maximum AFPS of 35.12% when compared to alternative models. Fig 11 depicts the accuracy performance analysis for the Brackish and URPC 2020 dataset.

**Fig 12:**
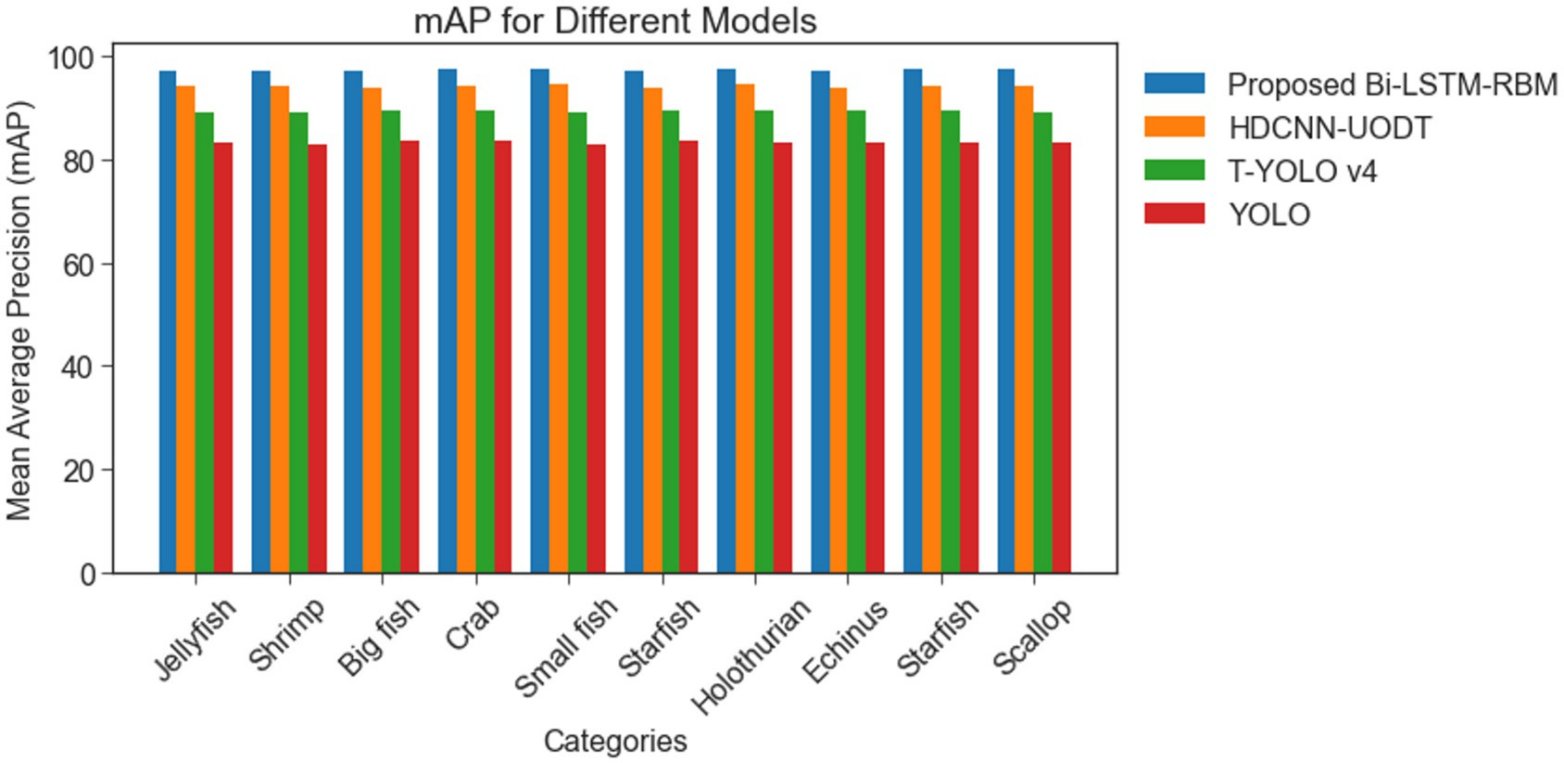
mAP comparative analysis for brackish dataset and URPC 2020 dataset.

Fig 12 depicts the mean average precision of Brackish and URPC 2020 dataset. The findings suggest that the UOD model utilizing BI-LSTM-RBM has yielded a greater mean average precision (mAP) than alternative methodologies, including HDCNN-UOD, T-YOLO v4, and YOLO. The BI-LSTM-RBM-based UOD model has demonstrated a notable improvement in the mAP on the Brackish dataset, achieving a value of 97.15%. In contrast, alternative methodologies have achieved diminished mAP scores, with 94.07%, 89.93%, and 83.13%. Based on the results, it can be observed that the BI-LSTM-RBM-based UOD model represents a proficient approach for detecting underwater objects. This can be attributed to its relatively low computational cost and processing time.

**Fig 13:**
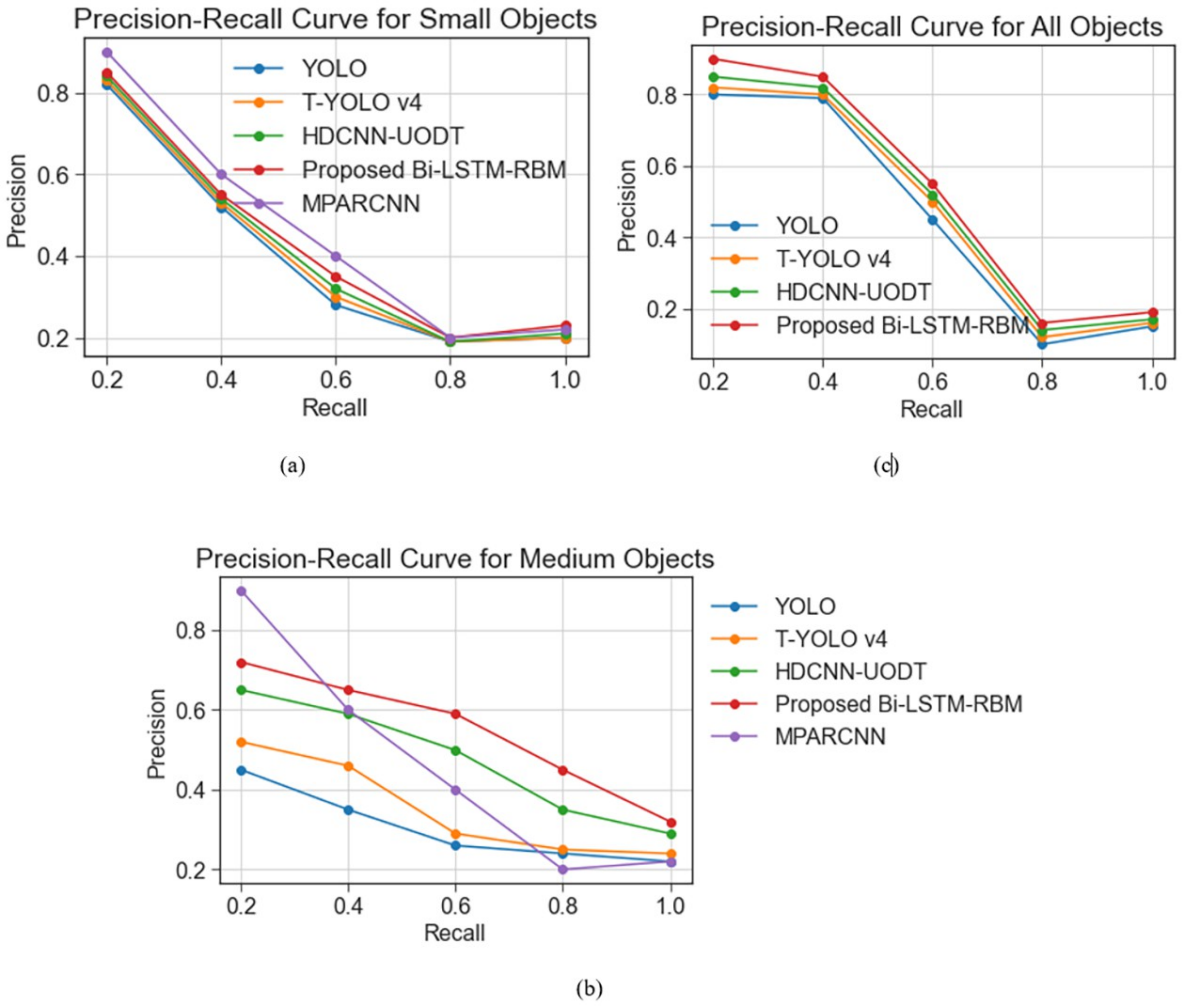
P-R curves for URPC 2020 dataset.

Fig 13 illustrates the pressure-response curves for detecting large, medium, and small objects and all objects in the URPC 2020 dataset. The method under consideration can attain superior results concerning the detection of objects comprising diverse scales. The method employed for detecting small objects yields a significantly higher p-r curve than other available detection networks, particularly in instances where the recall rate ranges from 0.6 to 1. The present findings suggest that the proposed approach exhibits superior performance regarding the identification of objects at multiple scales in subaquatic images with poor visual quality. Notably, the most significant enhancement of detection precision is achieved for objects characterized by relatively small dimensions compared to alternative state-of-the-art techniques. In general, this proposed method serves as a highly efficient and expeditious means of detecting underwater targets of various scales. It is notable for striking a favourable equilibrium between its detection accuracy and speed.

**Fig 14:**
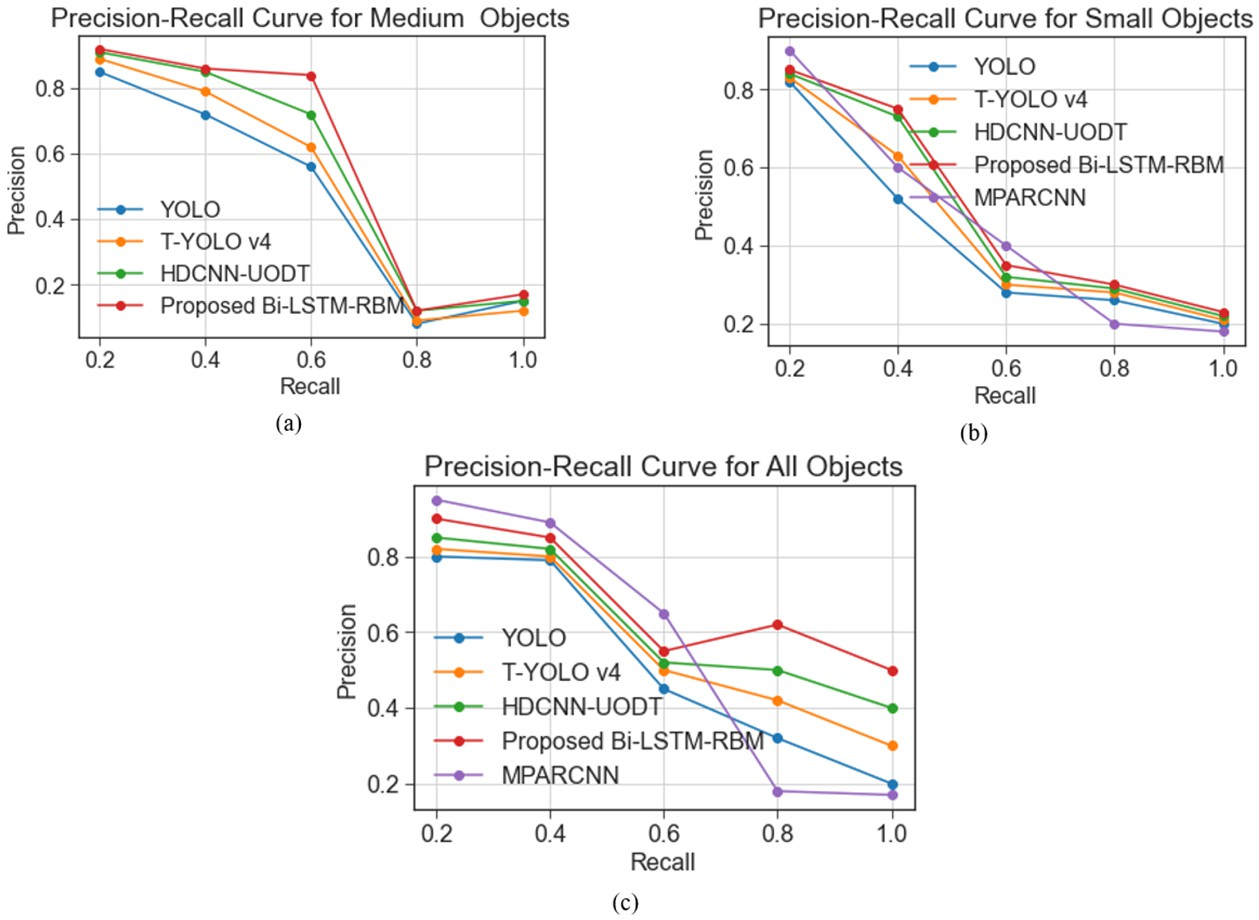
P-R curves for brackish dataset.

Fig 14 depicts the p-r curves for identifying objects of varying sizes, i.e., small, medium, large, and all objects, on the URPC 2020 dataset. The research methodology yields optimal outcomes in the identification of objects differing in magnitude. Specifically, the study observed that when the recall rate ranges from 0.6 to 0.1, the object detection methodology’s precision-recall (p-r) curve surpasses the current detection networks.

This finding suggests that the efficacy of the approach in detecting multi-scale entities within low-quality underwater imagery is most notably enhanced concerning small-scale objects, as compared to alternative advanced methods. The insufficient size of the dataset, coupled with the homogeneity of the dataset images in terms of lighting and background, results in reduced detection accuracy when utilizing the trained model in varied sea regions or environmental conditions. To address this limitation, this method intends to capture additional underwater imagery in diverse sea areas and conditions, thereby pre-processing the dataset and enabling accurate underwater detection. The imperative of this research is to advance autonomous UOD in challenging deep-sea environments by proposing a hybrid Bi-LSTM and RBM approach that combines sequence processing and hierarchical feature learning to significantly enhance the precision of object detection in dynamic underwater scenarios, ultimately contributing to the development of more robust and efficient autonomous systems for deep-sea resource exploration and underwater tasks.

## 5. Conclusion

This research proposed a novel hybrid model for UOD using the integration of Bi-LSTM and RBM techniques. Initially, the data augmentation technique was employed to expand the training dataset’s magnitude. The research model was focused on extracting significant features with the CNN model. The CNN model was employed to extract elevated-level features from underwater objects to enhance detection accuracy. The features extracted using the CNN are subject to normalization procedure to ensure the accuracy of UOD performance, after which they are incorporated into the deep BiLSTM-RBM to acquire the temporal details necessary for identifying underwater objects. The research method involves processing critical segments, rather than the entire observation, to decrease the research model’s computational complexity. The normalization of CNN features is conducted before their actual processing, facilitating the recognition of Spatiotemporal information with ease. To evaluate the efficacy of the Bi-LSTM-RBM-based UOD technique, a sequence of simulations was conducted utilizing three established benchmark datasets, namely brackish and URPC 2020 dataset. A comprehensive comparative analysis indicated that the Bi-LSTM-RBM-based UOD technique exhibits superior performance compared to contemporary approaches, thus affirming its aptitude for employment in object detection applications. In the future, due to the restrictions of current small-scale databases, there will be a collective effort to augment accuracy through the application of more extensive databases. Additionally, advanced diffusion-based models, coupled with metaheuristic optimization methodologies, will be developed to refine the efficacy of detection performance.
